# Effect of Surface Defect States on Valence Band and Charge Separation and Transfer Efficiency

**DOI:** 10.1038/srep32457

**Published:** 2016-09-02

**Authors:** Juan Xu, Yiran Teng, Fei Teng

**Affiliations:** 1 Jiangsu Engineering and Technology Research Center of Environmental Cleaning Materials (ECM), Nanjing University of Information Science & Technology, 219 Ningliu Road, Nanjing 210044, China; 2 Jiangsu Collaborative Innovation Center of Atmospheric Environment and Equipment Technology (CICAEET), Nanjing University of Information Science & Technology, 219 Ningliu Road, Nanjing 210044, China; 3 School of Environmental Science and Engineering, Nanjing University of Information Science & Technology, 219 Ningliu Road, Nanjing 210044, China; 4 Jiangsu Joint Laboratory of Atmospheric Pollution Control (APC), Nanjing University of Information Science & Technology, 219 Ningliu Road, Nanjing 210044, China; 5 Jiangsu Key Laboratory of Atmospheric Environment Monitoring and Pollution Control (AEMPC), Nanjing University of Information Science & Technology, 219 Ningliu Road, Nanjing 210044, China

## Abstract

Both energy band and charge separation and transfer are the crucial affecting factor for a photochemical reaction. Herein, the BiOCl nanosheets without and with surface bismuth vacancy (BOC, V-BOC) are prepared by a simple hydrothermal method. It is found that the new surface defect states caused by bismuth vacancy have greatly up-shifted the valence band and efficiently enhanced the separation and transfer rates of photogenerated electron and hole. It is amazing that the photocatalytic activity of V-BOC is 13.6 times higher than that of BOC for the degradation methyl orange (MO). We can develop an efficient photocatalyst by the introduction of defects.

As a green advanced oxidation technology, solar energy-driven photocatalysis has attracted much attention^1^. However, its practical applications are still limited by low quantum efficiency, light utilization efficiency and photoactivity. Thus, it remains a big challenge to develop inexpensive, highly efficient photocatalysts for the practical applications.

Recently, considerable attention has been paid to the BiOCl photocatalyst, due to its exceptional optical and electronic properties, nontoxicity, low cost and high photocatalytic activity^2^,^3^,^4^,^5^. Due to a wide band gap (3.19–3.60 eV), nevertheless, BiOCl can only absorb ultraviolet light, which is merely about 3–5% of solar energy^6^. Consequently, many attempts have been made to solve the problem, for example, morphology control^7^, heterojunction^8^,^9^,^10^,^11^,^12^,^13^,^14^,^15^,^16^, doping with mental or non-metal^17^, etc. In the latter case, however, the bulk defects introduced by dopants can act as the recombination centres of electron-hole pairs, resulting in the decrease of photoactivity^18^. Compared with bulk defects, surface defects as charge traps can prevent the electron-hole recombination, thus contributing to the improvement of photocatalytic activity^19^,^20^. The effect of oxygen vacancies on photoactivity has also been widely reported by researchers^21^,^22^,^23^,^24^,^25^,^26^,^27^. For example, Zhu *et al*. have demonstrated that the introductions of surface oxygen vacancies in ZnO and BiPO_4_ are conductive to the narrowing of band gap and the improvements of photoactivity^28^,^29^,^30^. Liu *et al*. investigated the effect of surface cation vacancy on the photocatalytic activity of CoSe_2_ nanosheets^31^. Guan *et al*. have also reported the formation of triple Bi-O-Bi vacancy in BiOCl^32^. To the best of our knowledge, however, the effect of the BiOCl with surface bismuth defects on photoactivity has never been in detail reported so far.

Herein, we report the generation of surface bismuth defects of BiOCl nanosheets. It is amazing that the BiOCl nanosheets with surface bismuth defects (V-BOC) show an exceptionally higher photocatalytic activity, compared with the BiOCl nanosheets without surface bismuth defects (BOC). We have explored the essence relationship between surface bismuth defects and activity. This study may provide a new idea to develop efficient photocatalysts.

## Results and discussion

### Crystal structure of BOC and V-BOC

The crystalline structures of the BOC and V-BOC samples are determined by X-ray diffraction (XRD) (Figure S1, Seeing electronic supporting information, (ESI)). All the diffraction peaks of both samples can be well indexed to the tetragonal phase of BiOCl with the cell constants of *a* = 3.891 Å, *c* = 7.369 Å (JCPDS No. 06-0249), which has a PbFCl^−^ -type structure with a space group of *P4/nmm*^33^. No the other impurities peaks are detected, indicating the formation of the phase-pure BiOCl. The layered BiOCl is constructed by the Cl ion layer and the bismuth-oxygen (Bi-O) layer.

Figure S2 (ESI) shows the scanning electron microscopy (SEM) images of BOC and V-BOC samples. The BOC sample is composed of the microspheres with the diameters ranging from 4 to 7 μm (Figure S2a, ESI). The microspheres are assembled by numerous nanosheets with 0.3 μm × 0.5 μm × 0.2 μm (Figure S2b, ESI), which is similar to our previous report^34^. The V-BOC sample (Figure S2c,d, ESI) is composed of the nanosheets of 1.5 μm × 1 μm × 0.2 μm. Their high-resolution transmission electron microscopy (HRTEM) images are shown in Figure S3 (ESI) to clearly prove the presence of the defect shell. For BOC and V-BOC samples, the lattice fringe spacing is 0.34 nm, corresponding to the (101) plane of BiOCl. Compared with Figure S3a (ESI), we can see that the thickness of defect shell is about 5.51 nm from Figure S3b (ESI), and the lattice fringes of V-BOC become indiscernible indicated by red circle (Figure S3c,d, ESI). This indicates that the defects exist everywhere in the V-BOC sample.

### XPS, ICP and FT-IR spectra

**Figure 1:**
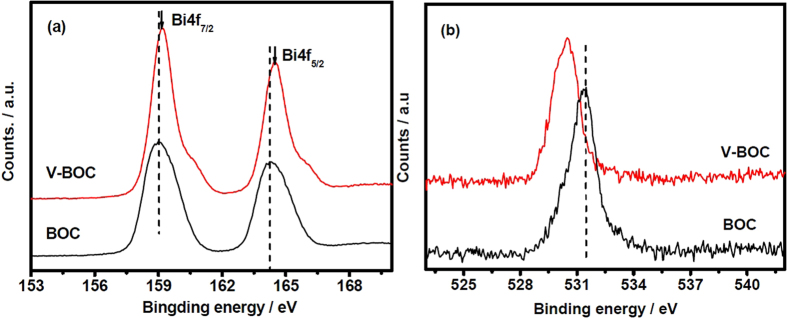
(**a**) Bi 4f and (**b**) O1s photoelectron spectra for the BOC and V-BOC samples.

To further understand the defect structure, the XPS surface elements analysis is performed to detect the Bi and O atoms in both BiOCl samples. In Table 1, the atom ratio of Bi to O is 0.72 for the V-BOC sample, which is slightly lower than that (0.75) of BOC. Moreover, the inductively coupled plasma mass spectrometry (ICP-MS) has been performed to detect the Bi and O in both BiOCl samples. The ICP-MS results (Table 2) show that the Bi/O ratio in BOC and V-BOC are 0.85 and 0.81, respectively. This also suggests that the bismuth vacancies are generated in the V-BOC sample. As a surface analysis method, the XPS analysis is mainly focused on the surface atoms layer with the depth about 2–5 nm for inorganic semiconductor materials, which fits well thickness of defect shell (Figure S3, ESI). The survey XPS spectra of both samples are shown in Figure S4 (ESI). We can observe that the elements present are Bi, O, C and Cl, the peak at 284.8 eV could be readily assigned to the binding energies of C 1 s (Figure S4, ESI), which results from the adventitious carbon on the sample surface that is nearly unavoidable. This also indicates the formation of the pure BiOCl. Figure 1 shows their Bi 4f and O1s peaks. Compared with BOC, the Bi 4f peaks of V-BOC shift to a higher binding energy (Fig. 1a). Furthermore, the binding energy of O 1s is about 530.4 eV for V-BOC, which is smaller than that (531.5 eV) of BOC (Fig. 1b). This shift may be attributed to the weakened hybridization between Bi 6s and O 2p, which leads to the decrease of binding energy of bismuth-oxygen bond (Bi-O)^30^. These shifts indicate the formation surface bismuth defects or oxygen vacancies in the V-BOC^35^. From Table 1 and Table 2, we can see the O atom of V-BOC is the same with the BiOCl. So the formation of defect in the V-BOC sample is attributed to the lack of Bi element. The same phenomenon has occurred in Bi_6_S_2_O_15_ system^30^, in which they have reported that the generation of surface bismuth defects resulted in a lower binding energy^30^.

The Fourier transform infrared (FT-IR) spectra show the characteristic absorption peaks of these two samples (Figure S5, ESI). The absorption bands at 1400 cm^−1^ could be assigned to the asymmetry and symmetric stretching vibrations of Bi-Cl band, while the absorption bands at 522 cm^−1^ are assigned to the Bi-O stretching vibrations^36^. The bands at 3455 and 1623 cm^−1^ are the stretching and flexural vibrations of O-H for adsorbed water molecules, respectively^36^. The bands at 2927 and 1043 cm^−1^ stem from the stretching vibrations of C-H and C-O-C bonds, respectively; which are originated from ethylene glycol molecules absorbed on the surface of the samples^37^. No significant change in FT-IR spectra can be found for both samples, indicating that the crystal structure does not change, which is in good agreement with the XRD results.

### Optical property

**Figure 2:**
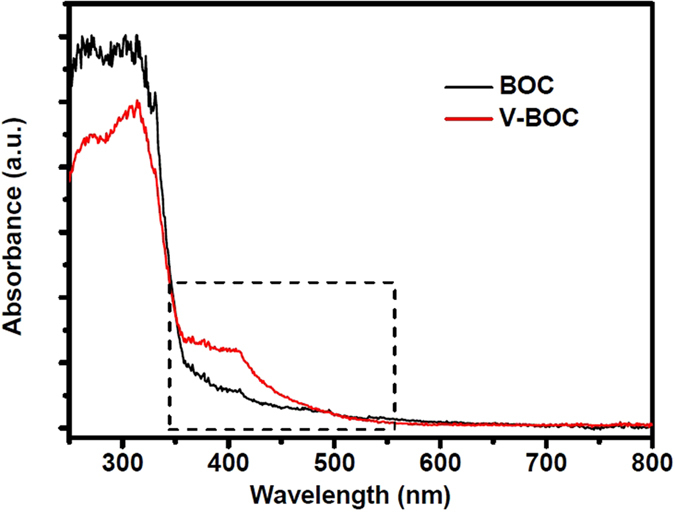
UV-vis diffuse reflectance spectra (UV-DRS) of the BOC and V-BOC samples.

Figure 2 displays the ultraviolet-visible diffuse reflection spectra (UV-DRS) of both samples. It is interesting that the UV-DRS spectral response of V-BOC sample is greatly expanded and enhanced because of the introduction of surface bismuth vacancy. The absorption edge of BOC is about 381.5 nm, corresponding to the band gap of 3.25 eV. In comparison, the main absorption edge and the highest absorption peak of V-BOC nanosheets do not change (λ < 355 nm). However, its absorbance is enhanced obviously in the range 350–500 nm, the band gap of the V-BOC nanosheets is ~2.91 eV, which may be caused by surface bismuth vacancy, as it does in Bi_6_S_2_O_15_^30^. The improved optical absorption is expected to contribute to the improvement of photocatalytic activity.

### EPR spectra

**Figure 3:**
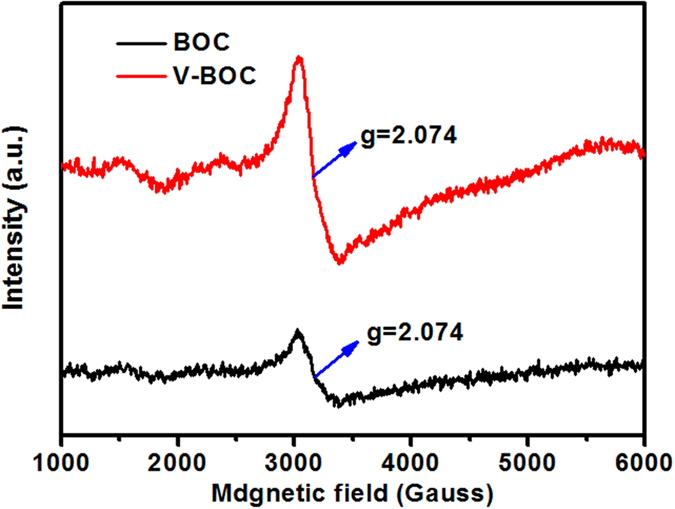
EPR spectra of the BOC and V-BOC samples.

Moreover, electron paramagnetic resonance (EPR) of the as-prepared samples has been performed (Fig. 3). EPR is a sensitive and direct technology to monitor surface defect behaviors of material. From Fig. 3, it can be found that for V-BOC, the intensity of the EPR signal at *g* factor of ~2.074 is much higher than that of BOC. The peak at *g* = ~2.1 has been reported previously to be the surface bismuth defects^38^. This result is consistent with the valence band based X-ray photoelectron spectroscopy (XPS) analysis. Both XPS and EPR results demonstrate the presence of the surface Bi vacancy in V-BOC.

### Valence band based XPS and Mott-schottky plots

The UV-DRS (Fig. 2) reveals that the band gap of the V-BOC nanosheets is ~2.91 eV, smaller than that (3.25 eV) of BOC, which can be attributed to the existence of surface bismuth defects.

**Figure 4:**
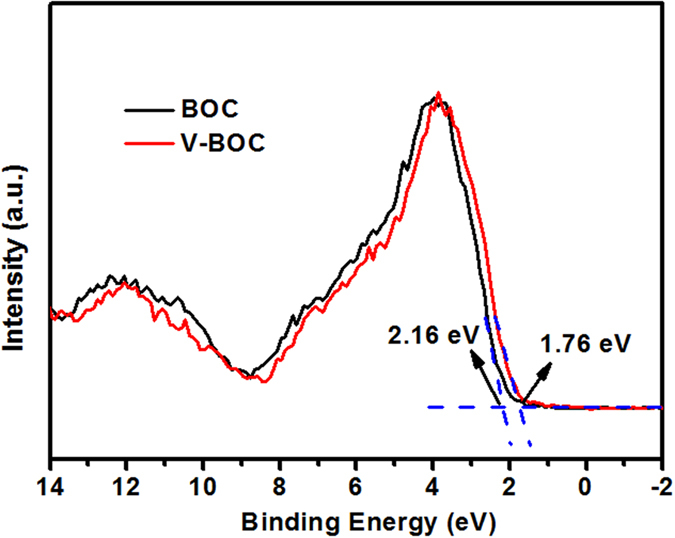
Valence-band XPS spectra of the BOC and V-BOC samples.

Further, the valence bands (VB) of both BOC and V-BOC are measured by the valence band X-ray photoelectron spectroscopy (XPS) to confirm the energy band positions, as shown in Fig. 4. BOC displays a VB with the edge of the maximum energy at about 2.16 eV. Combining the test results of UV-DRS (Fig. 2), the CB minimum energy is −1.09 eV. For V-BOC, however, the VB maximum energy up shifts to 1.76 eV, compared with that of BOC. Combined with the results of UV-DRS (Fig. 2), the CB minimum of V-BOC occurs at −1.15 eV and shifts up by 0.06 eV, compared with that of BOC. According to the results above, the introduction of surface bismuth vacancies in our research elevated whole bands and many shallow surface bismuth-vacancy states appear above and partly overlapping with the VB. And the valence-band maximum (VBM) of V-BOC is raised by 0.4 eV. We could draw a conclusion that the VB width was widen due to the presence of the surface Bi vacancy.

Furthermore, the Mott-Schottky plots are also tested to confirm the energy band positions, the CB bottoms of BOC and V-BOC can be estimated by the Mott-Schottky plots. It can be calculated through the following Equation (1):


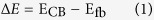


where E_fb_ is the flat-band potential, E_CB_ is the conduction band potential. Herein, Δ*E* is assumed to be −0.3 V for the *n*-type semiconductor. As shown in Figure S6, the E_fb_ are −0.37 and −0.84 V *vs*. SCE for BOC and V-BOC, respectively. As a result, the CB potential of V-BOC is about −1.14 V *vs*. SCE, which is higher than that (−0.67 V *vs*. SCE) of BOC. Combining with the determined band gap by UV-DRS, the oxidation potentials of VB are estimated to be 2.58 and 1.77 V *vs*. SCE for BOC and V-BOC, respectively.

### Photochemical properties

**Figure 5:**
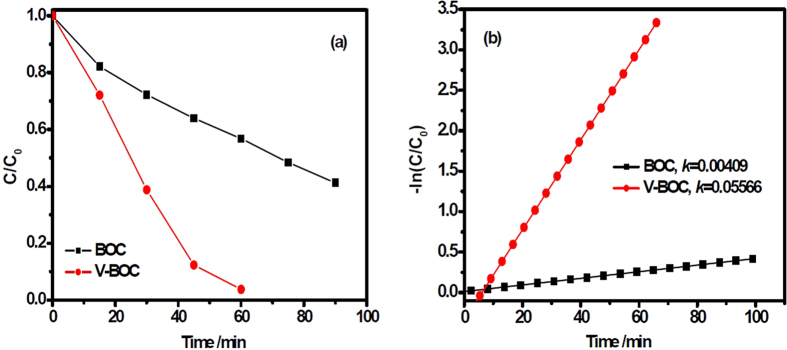
(**a**) Degradation curves and (**b**) reaction kinetic curves of MO over the BOC and V-BOC samples under UV light irradiation (λ ≤ 420 nm).

Their photocatalytic activities are evaluated by the degradation of methyl orange (MO) under visible light (λ > 420 nm) and UV light irradiation (λ ≤ 420 nm). Although the V-BOC can absorb visible light, MO can not be degraded under visible light (λ > 420 nm) (Figure S7, ESI). As shown in Fig. 5a, it is clear that the V-BOC shows a greatly higher photocatalytic activity than BOC. The apparent reaction rate constants are determined to be 0.00409 and 0.05566 min^−1^ for BOC and V-BOC, respectively (Fig. 5b). The photocatalytic activity of V- BOC is about 13.6 times as high as that of BOC. Further, we have carried out cycling experiments to investigate the stability of V-BOC (Figure S8, ESI). The photocatalytic activity of V-BOC does not decrease after 4 cycles, demonstrating that the V-BOC photocatalyst is stable.

It is well known that the photocatalytic activity of photocatalyst is affected by various factors, such as crystal structure, particle morphology, surface area, band gap, separation and transfer efficiency of charges, and so on^39^,^40^. First, the crystal phases are same for both samples. Second, the BOC sample is composed of the microspheres with the diameters ranging from 4 to 7 μm, and the microspheres are made up of numerous nanosheets with the size of 0.3 μm × 0.5 μm × 0.2 μm. The size of BOC nanosheets is much smaller than that (1.5 μm × 1 μm × 0.2 μm) of V-BOC. Moreover, the BET area (15.9 m^2^ g^−1^) of the BOC sample is much larger than that (4.3 m^2^ g^−1^) of V-BOC sample. On base of BET areas, the BOC should have a higher activity for the degradation of MO than that of V-BOC. However, the actual test result is that V-BOC has a greatly higher activity than BOC, indicating that the particle morphology is not the main factor. The results imply the enhanced activity of V-BOC has nothing to do with these two factors. Third, the separation and transfer efficiency of electron-hole pairs are investigated by the electrochemical impedance spectra (EIS)^41^. In Figure S9a (ESI), the arc radius of V-BOC is smaller than that of BOC. A smaller semicircle radius in EIS Nyquist plot means a smaller electric resistance of electrode, thus the former has a higher electron transfer ability than the latter.

### PL and photocurrent spectra

Moreover, the separation efficiency of the photo-generated electrons and holes can be investigated by photoluminescence (PL)^42^. Figure S9b (ESI) shows their PL spectra under the excitation wavelength of 381 nm. Both samples show the intrinsic fluorescence emission peaks at around 381 nm. The peak intensity of the V-BOC is lower than that of BOC. Generally, a low PL emission intensity indicates a low recombination efficiency of photogenerated charges, leading to a high photocatalytic activity.

Further, from Figure S9c (ESI) shows the photocurrent response spectra at each switch-on and switch-off event for both electrodes, in which the photocurrent response of V-BOC is higher than that of BOC. The result is in accord with the photocatalytic activity order. The higher photocurrent response of V-BOC indicates the improved separation efficiency of photoinduced electron-hole pairs, which could be caused by the induction of the surface bismuth vacancy. We assume that the surface bismuth vacancy, like conventional oxygen vacancy^37^, may serve as the carrier trap to prevent the electrons and holes recombination. Summarily, it is the surface bismuth vacancy with higher electronic conductivity that improves the charge separation efficiency, eventually enhancing the photocatalytic activity.

### Trapping experiments

To further elucidate the photocatalytic mechanism, the trapping experiments are performed to detect the main oxidative species (radicals or holes) in the photocatalytic process, in which (NH_4_)_2_C_2_O_4_∙H_2_O as hole scavenger and isopropyl alcohol (IPA) as hydroxyl radical scavenger are added to the reaction system during the degradation of MO, respectively (Figure S10, ESI). In both BOC- and V-BOC-containing reaction systems, the degradation activities of MO are both greatly reduced while adding (NH_4_)_2_C_2_O_4_∙H_2_O. On the contrary, the addition of IPA only causes a small change in the degradation of MO. Thus, the results suggest that the holes are the main oxidative species in both systems.

### Role of surface vacancy states

**Figure 6:**
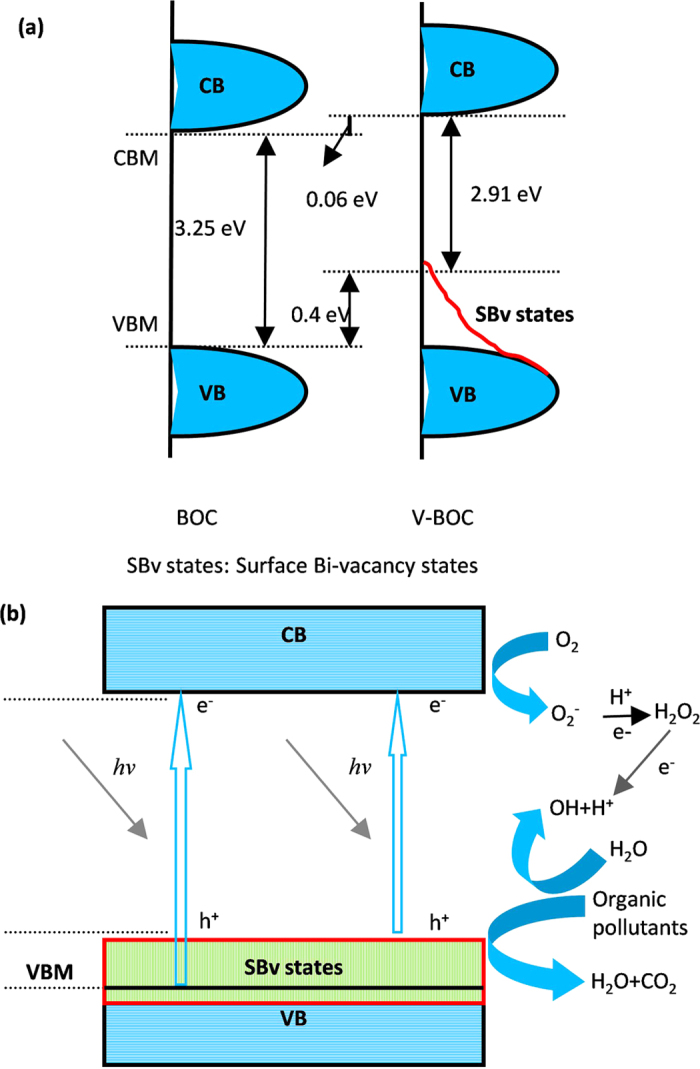
Schematic diagram of (**a**) the positions of VB and CB of BOC and V-BOC; (**b**) the reaction mechanism over BOC and V-BOC under UV light irradiation (λ ≤ 420 nm).

On Base of the results above, we have proposed a schematic diagram to describe the energy band position variation of V-BOC (Fig. 6a). Also, a mechanism of charge separation and photocatalytic reaction of V-BOC is shown in Fig. 6. The forbidden band of BiOCl is wide (about 3.25 eV), which can only be excited by UV light with the wavelengths shorter than 381.5 nm. After surface bismuth vacancies are introduced, however, the shallow surface bismuth-vacancy states (SBv. states) are generated, one part of which locates above, and another part of which overlaps with the VB of BiOCl. This would narrow the band gap, thus expanding the photo-response wavelength to 426.1 nm (about 2.91 eV). Although the V-BOC can absorb visible light, MO cannot be degraded under visible light (λ > 420 nm). We assume that the vacancy mainly contribute to the improvement of separation and transport efficiency of electron-hole pairs. The similar results have also been reported previously by Sun *et al*.^21^ and Zhu *et al*.^30^ The VB width was widen due to the presence of the surface Bi vacancy. Guan *et al*.^32^ and Liu *et al*.^43^ have demonstrated that the increased VB width is beneficial for the separation of charge carriers, because the VB width intrinsically controls the mobility of holes: the wider the VB is, the higher the mobility of holes generated is^32^,^43^. The VB width not only makes the better photo-oxidation of holes, but also promotes the transfer of photoexcited electrons to reactants, favoring for the inhibition of the electron-hole recombination^32^,^43^. Thus the widened VB width can improve the separation and transport of electron-hole pairs.

On base of the energy band structure (Fig. 6b), we assume that O_2_ can be reduced to ∙O_2_^−^ by the photogenerated electrons, which could further transform to H_2_O_2_ via ∙O_2_^−^ + e + 2H^+^ → H_2_O_2_^44^. Besides, H_2_O is oxidized to ∙OH by the photogenerated holes. The formed ∙OH and holes are strong oxidants for dye molecules. Our results above have confirmed that holes are the main oxidative species, so the MO dye can be easily decomposed.

Summarily, the SBv. states by surface bismuth vacancy can not only narrow the band gap, but also widen the VB width, which contributes to the increase of light absorption range and the improvement of separation and transport efficiency of photoinduced electron-hole pairs, thus the photocatalytic activity can be improved by surface bismuth vacancy.

## Conclusions

To conclude, the surface bismuth vacancies have greatly up shifted the valence band (VB), and narrowed the band gap of V-BOC. Thus, the photocatalytic activity of V-BOC has been greatly improved. This work provides us with a powerful strategy to develop a highly-efficient photocatalyst.

## Methods

All reagents were of analytical grade, purchased from Beijing Chemical Reagents Industrial Company of China, and were used without further purification.

### Synthesis of BiOCl nanosheets without surface bismuth vacancy (BOC)

The BiOCl nanosheets without surface bismuth vacancy (BOC) was prepared according to previously reported method^34^. In detail, 1.5 mmol of Bi(NO_3_)_3_·5H_2_O was dissolved in 30 mL of ethylene glycol (EG) under stirring at room temperature. After the Bi(NO_3_)_3_·5H_2_O was completely dissolved, 10 mmol of NaCl was added to the above solution. After stirring for 30 min, the solution was transferred into a 50-mL Teflonlined stainless steel autoclave, and then maintained at 170 °C for 6 h. After reaction, the autoclave was cooled to room temperature naturally. The resulting precipitate was centrifuged and washed with ethanol and distilled water three times, and then dried at 60 °C for 3 h.

### Synthesis of BiOCl nanosheets with surface bismuth vacancy (V-BOC)

In a typical procedure, 0.5 mmol Bi_2_O_3_ and 8 mmol NH_4_Cl (1:16 molar ratio) were mixed together, and then 20 mL of H_2_O_2_ was added. The resulting precursor suspension was magnetically stirred for 30 min at room temperature and then transferred into 50-mL Teflon-lined stainless steel autoclave, and then maintained at 180 °C for 12 h. After reaction, the autoclave was cooled to room temperature naturally. The resulting precipitate was centrifuged and washed with ethanol and distilled water three times, and then dried at 60 °C for 3 h.

### Sample characterizations

The crystal structures of the samples were determined by X-ray powder polycrystalline diffractometer (Rigaku D/max-2550 VB), using graphite monochromatized Cu K radiation (λ = 0.154 nm), operating at 40 kV and 50 mA. The XRD patterns were obtained in the range of 20–80^◦^ (2θ) at a scanning rate of 7^◦^ min^−1^. The samples were characterized on a scanning electron microscope (SEM, Hitachi SU-1510) with an acceleration voltage of 15 keV. The samples were coated with 5-nm-thick gold layer before observations. The fine surface structures of the samples were determined by high-resolution transmission electron microscopy (HRTEM, JEOL JEM-2100F) equipped with an electron diffraction (ED) attachment with an acceleration voltage of 200 kV. Nitrogen sorption isotherms were performed at 77 K and <10^−4^ bar on a Micromeritics ASAP2010 gas adsorption analyzer. Each sample was degassed at 150 ^o^C for 5 h before measurements. Surface area was calculated by the Brunauer-Emmett-Teller (BET) method. Fourier transform infrared spectra (FT-IR) were recorded on a Fourier transform infrared spectra (FT-IR, KBr disk method; Thermo Scientific Nicolet iS5) at the wavenumber range of 400–4000 cm^−1^.

X-ray photoelectron spectroscopy (XPS) measurements were done on a VG ESCALAB MKII XPS system with Mg K_α_ source and a charge neutralizer. All the binding energies were referenced to the C1s peak at 284.8 eV of the surface adventitious carbon. The electron paramagnetic resonance (EPR) spectra were collected using a Bruker ESP 500 spectrometer at 90 K. UV-vis diffused reflectance spectra (UV-DRS) of the samples were obtained using a UV-vis spectrophotometer (UV-2550, Shimadzu, Japan). BaSO_4_ was used as a reflectance standard in a UV-vis diffuse reflectance experiment. The valence band based X-ray photoelectron spectroscopy (XPS) was performed to estimate the VB position of the as-prepared samples through a PHI 5300 ESCA system. The photoluminescence (PL) spectra were obtained on Cary Eclipse fluorescence spectrophotometer at room temperature. The PL lifetime was measured using time-resolved fluorescence decay spectra by time-correlated single-photon counting under the 260 nm laser excitation (Cary Eclipse, Agilent). The Mott-Schottky method was applied to measure the flat potential (E_fb_) of semiconductor particle films, which are immersed in 0.1 mol L^−1^ Na_2_SO_4_ solution (pH = 7), which is carried out in conventional three electrode cells using a CHI660D electrochemical workstation (Shanghai Chenhua Instrument Co., Ltd., Shanghai, China).

### Measurements of photocurrents and EIS

An electrochemical system (CHI-660B, China) was employed to measure the photocurrents and electrochemical impedance spectroscopy (EIS). Electrochemical impedance spectroscopy (EIS) was performed from 0.1 Hz to 100 kHz at an open circuit potential of 0.3 V and an alternating current (AC) voltage amplitude of 5 mV. The data were analyzed by ZSimWin software. Photocurrent measurements were carried out in a conventional three-electrode system, in which indium-tin oxide (ITO) glass was used as the current collector to fabricate photo electrode, and 0.1 M Na_2_SO_4_ was used as the electrolyte solution. BOC/ITO and V-BOC/ITO photo electrode were prepared by a coating method. Potentials were given with reference to the standard calomel electrode (SCE).

### Evaluation of photo catalytic activity

The photo catalytic activity of the sample was evaluated by the degradation of methyl orange (MO) aqueous solution under UV light (λ ≤ 420 nm), using a 300 W Xe arc lamp (CEL-HXF 300) equipped with an ultraviolet cutoff filter as a light source. The reaction system was placed in a sealed black box with the top opened, and was maintained a distance of 15 cm from the light source. The photocatalysts (100 mg) were dispersed in 200 mL of 10 mg/L MO aqueous solution in a Pyrex beaker at room temperature. Before lighting on, the suspension was continuously stirred for 30 min in the dark to ensure the establishment of an adsorption–desorption equilibrium between the catalysts and MO solution. During degradation, 3 mL of solution was collected by pipette at an interval of irradiation, and subsequently centrifuged to remove the catalysts. UV-vis absorption spectra were recorded on a Spectrumlab 722sp spectrophotometer to determine the concentration of MO. The degradation reaction could be expressed by an apparent first-order rate constant (*k*_*a*_), which could be calculated using the following Equation (2):


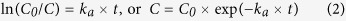


where *C*_*0*_ is the initial concentration of MO solution, and *C* is the concentration of MO at t-min irradiation, respectively. The active species generated in the photocatalytic reaction were detected through trapping experiments, in which 0.25 mmol isopropanol (IPA) and 1 mmol ammonium oxalate ((NH_4_)_2_C_2_O_4_) were used as hydroxyl radicals scavenger and holes scavenger, respectively.

## Additional Information

**How to cite this article**: Xu, J. *et al*. Effect of Surface Defect States on Valence Band and Charge Separation and Transfer Efficiency. *Sci. Rep.* **6**, 32457; doi: 10.1038/srep32457 (2016).

## Supplementary Material

Supplementary Information

## Figures and Tables

**Table 1:** XPS surface element analysis of the BOC and V-BOC samples.

Sample	Bi atom%	O atom%	Cl atom%	Bi:O
BOC	22.588	29.923	23.572	0.75:1
V-BOC	21.426	29.694	23.108	0.72:1

**Table 2:** Elemental composition of the BOC and V-BOC samples by inductively coupled plasma mass spectrometry (ICP-MS).

Sample	Bi atom%	O atom%	Cl atom%	Bi:O
BOC	24.783	29.156	29.026	0.85:1
V-BOC	23.866	29.385	31.866	0.81:1
