# Supplementary material for: Effect of Surface Defect States on Valence Band and Charge Separation and Transfer Efficiency
Source: Sci Rep. 2016 Sep 2;6:32457. doi: 10.1038/srep32457 (PMC5009424; doi:10.1038/srep32457)
Supplement: Supplementary Information [file srep32457-s1.pdf]

Supporting information for

Effect of Surface Defect States on Valence Band and Charge Separation and Transfer Efficiency

Juan Xu, Yiran Teng, Fei Teng*

Jiangsu Engineering and Technology Research Center of Environmental Cleaning Materials (ECM), Jiangsu Key Laboratory of Atmospheric Environment Monitoring and Pollution Control (AEMPC), Jiangsu Joint Laboratory of Atmospheric Pollution Control (APC), Collaborative Innovation Center of Atmospheric Environment and Equipment Technology (AEET), School of Environmental Science and Engineering, Nanjing University of Information Science & Technology，219 Ningliu Road, Nanjing 210044, China.

* Corresponding author. Tel./Fax.: +86-25-58731090; Email address: tfwd@163.com

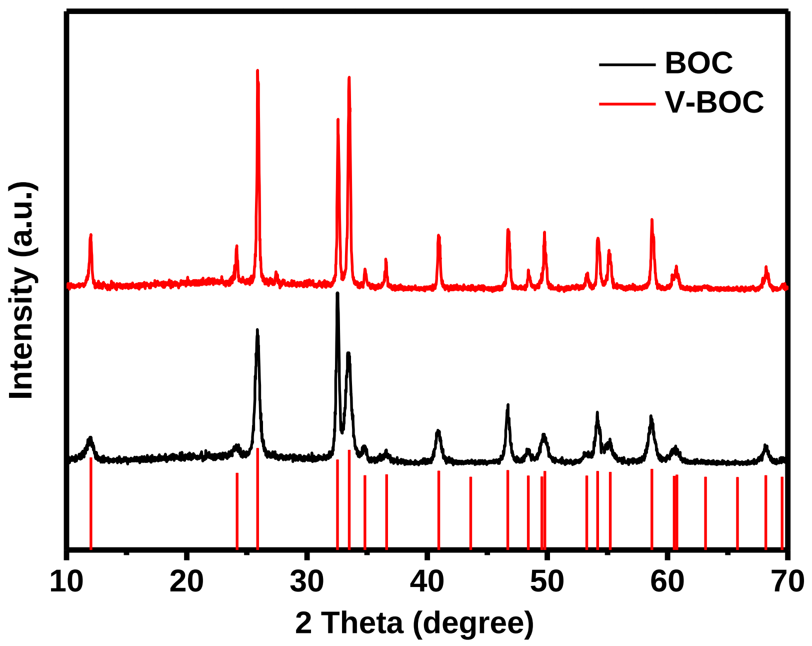

Fig. S1. X-ray diffraction (XRD) patterns of the BiOCl nanosheets with and without surface bismuth defects (V-BOC and BOC)

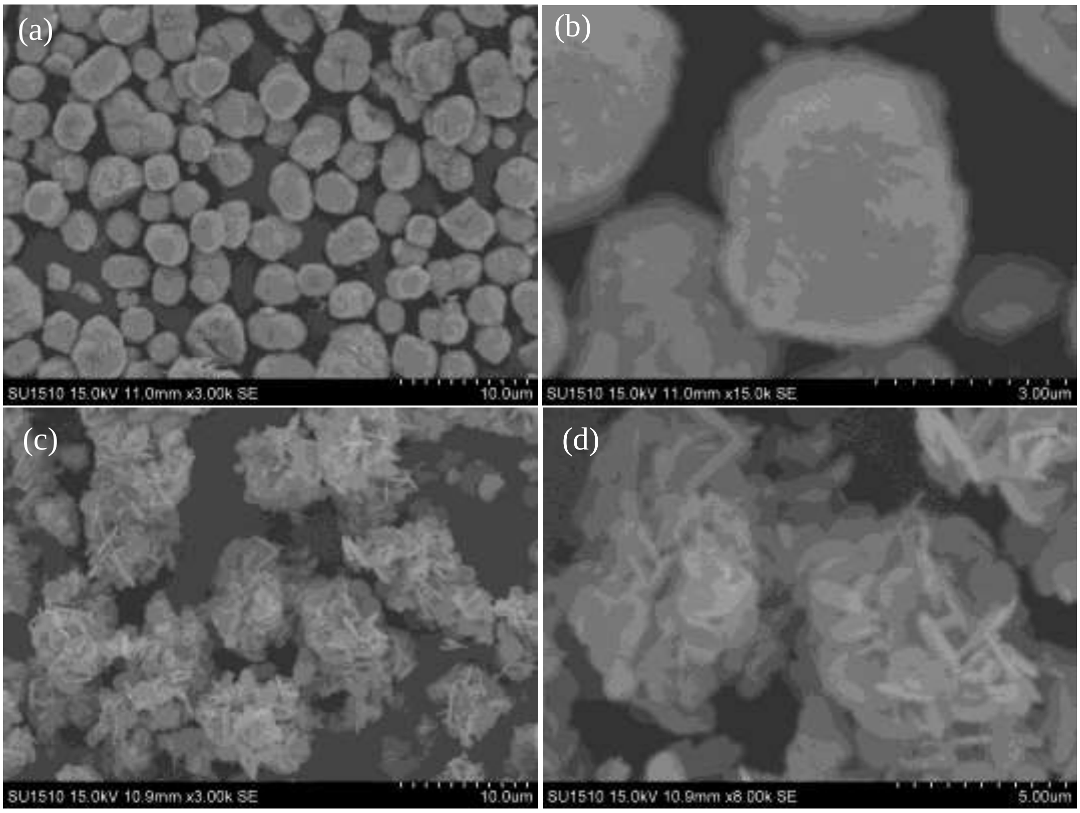

Fig. S2. SEM images of (a,b) BiOCl nanosheets without surface bismuth vacancy (BOC) and (c,d) BiOCl nanosheets with surface bismuth vacancy (V-BOC).

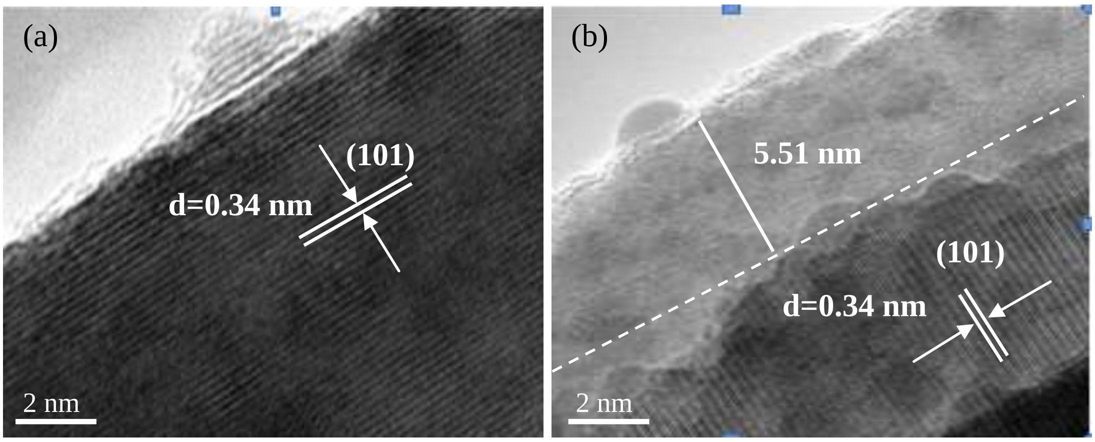

Fig. S3. HRTEM images of BOC (a) and V-BOC (b) samples

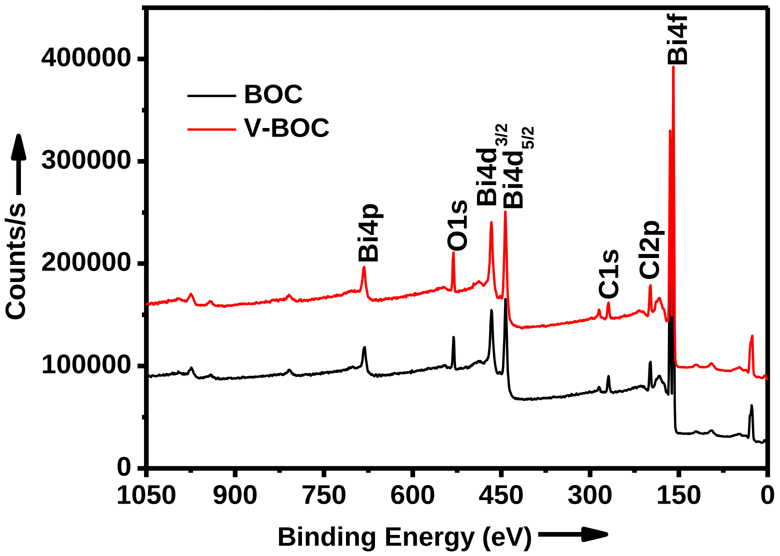

Fig. S4. Survey XPS spectrum of BOC and V-BOC samples

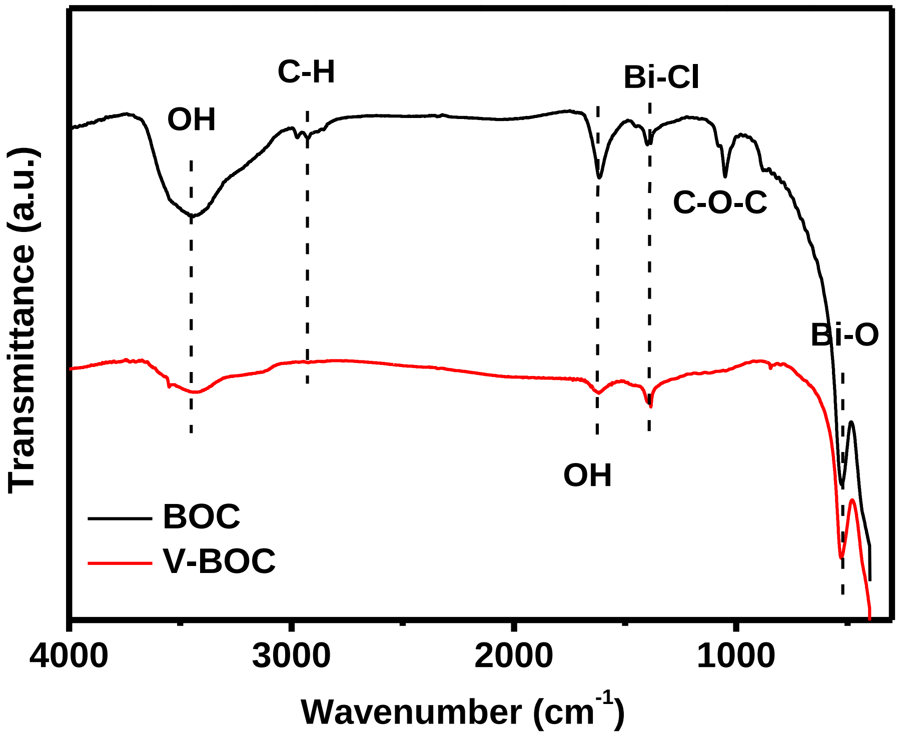

Fig. S5. FT-IR spectrum of BOC and V-BOC samples

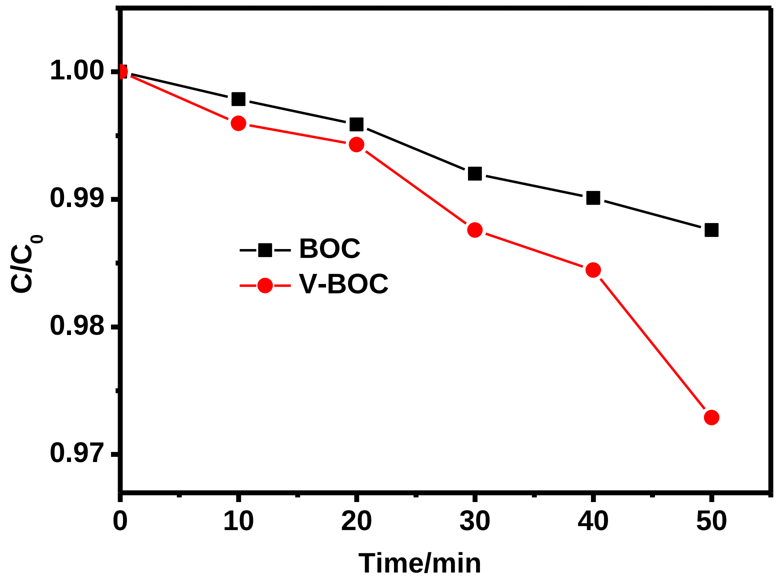

Fig. S6. Degradation curves of MO over the BOC and V-BOC samples under visible light irradiation (λ>420 nm)

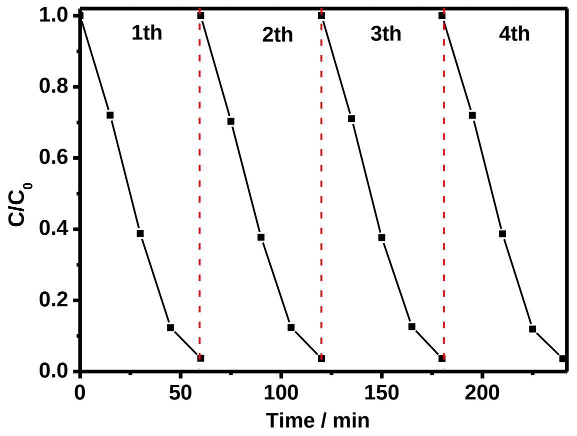

Fig. S7. Cycling stability of V-BOC for the photodegradation of MO under UV light irradiation (λ≤420 nm)

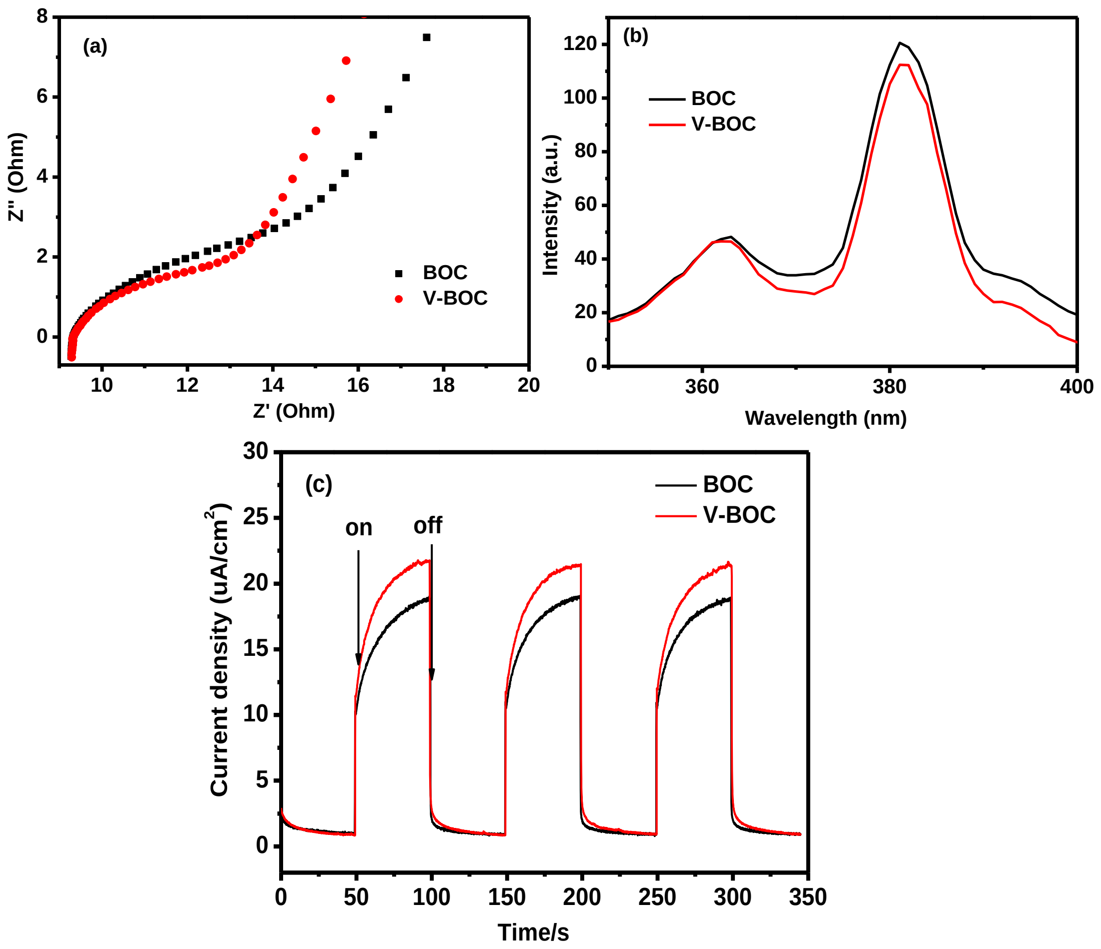

Fig. S8. (a) Electrochemical impedance spectroscopy (EIS) Nynquist plots, (b) PL emission spectra and (c) photocurrents of BOC and V-BOC nanosheets electrodes under UV light irradiation (λ≤420 nm)

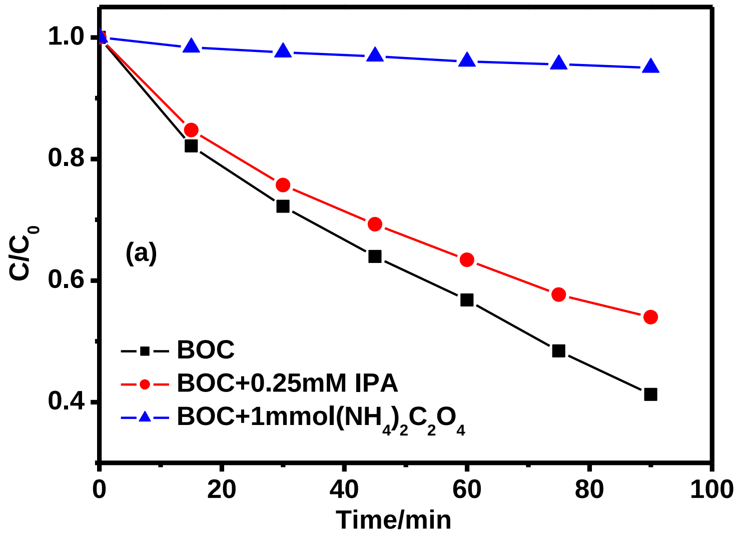

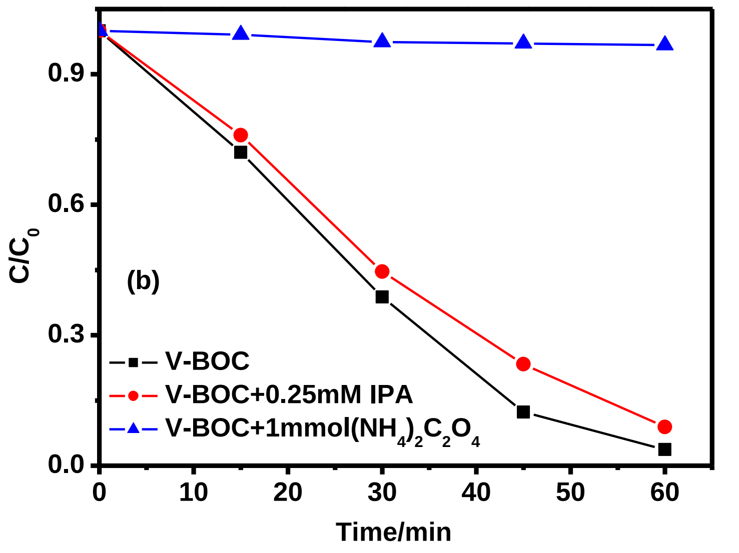

Fig. S9. The trapping experimental results for the degradation of MO over (a) BOC and (b) V-BOC under UV light irradiation (λ≤420 nm)
